# Physicochemical Characteristics of Biochar from Waste Cricket Chitin (*Acheta domesticus*)

**DOI:** 10.3390/molecules27228071

**Published:** 2022-11-21

**Authors:** Krzysztof Różyło, Katarzyna Jędruchniewicz, Patrycja Krasucka, Wojciech Biszczak, Patryk Oleszczuk

**Affiliations:** 1 Department of Herbology and Plant Cultivation Techniques, University of Life Sciences in Lublin, 20-033 Lublin, Poland; 2 Department of Radiochemistry and Environmental Chemistry, Faculty of Chemistry, Maria Curie-Skłodowska University, 20-033 Lublin, Poland

**Keywords:** cricket, chitin, biochar, biopolymer, waste management, phytotoxicity, amorphous carbon, FTIR spectra

## Abstract

The properties of biochar (BC) from crustacean chitin are relatively well understood, while there are few studies on BC from insect chitin. This study presents the characterization and phytotoxic assessment of BC produced from crickets and cricket chitin. Cricket powder (BCCR) and cricket chitin (BCCH) were pyrolyzed at 500 °C and 700 °C. Physicochemical characteristics, N ad-/desorption, FTIR, were examined. SEM images were also performed. Regardless of the pyrolysis temperature, biochars were characterized by a densely “packed” solid surface/monolithic type with a non-porous structure (0.05–0.22 m^2^/g) and high content of N (9.4–11.8%). BCCHs showed a higher pH (12.2–12.4) compared to BCCR (8.7–10.8). Based on the XRD analysis, BCs were characterized by an amorphous carbon turbostratic structure and a randomly oriented graphitic-like micro-crystallite structure. FTIR spectra of BCs confirmed the presence of various O_2_ and N-functional groups on the BC surface. BCCHs added to soil at rates from 0.5 to 1.5% significantly reduced the germination of *Lepidium sativum*. Stimulation of root elongation was also observed in the case of BCCR500 1.0% and BCCR700 1.5%. Thermal degradation of cricket powder and cricket chitin promotes the formation of organic N-containing heterocyclic rings, which lead to the production of N-doped carbons with potential uses in energy storage and the contaminations sorption.

## 1. Introduction

The bioconversion of plant foods to animal foods for human beings has a low energy efficiency [1,2,3]. This, combined with an increasingly global human population and increasing animal protein consumption, creates many environmental and economic problems [4,5,6]. Therefore, it is necessary to look for more efficient protein sources for livestock or directly for human beings. The breeding of insects including crickets (*Acheta domesticus*) is one of the directions of the search for alternative and efficient food sources for animals and human beings [7,8,9]. In Europe, insect protein production was about 1.9 thousand tons in 2018 and increased to 194 thousand tons in 2020. By 2025, a total of 1.2 million tons is expected [10].

Depending on the species, insect dry matter contains 23–76% protein, 10–59% fat, 2–16% fiber in the form of chitin [3]. The separated protein and fat are the raw material in the food industry and chitin is the waste. The total protein content of insects is potentially overestimated because a portion of it is chemically bound to the polysaccharide chitin that forms the exoskeleton. This relationship increases the real content of the waste chitin [11]. All the more, it is necessary to look for effective ways of utilizing and/or processing chitin derived from insects used for food purposes, which would be in line with the principles of waste management in the circular economy.

Chitin is a renewable, low cost and the second, after cellulose, most abundant natural biopolymer. It is obtained from the external exoskeleton of crustaceans, insects, as well as from cephalopods as the waste of the food industry and from some fungi (e.g., from the classis of *Saccharomyces*) [12,13,14,15]. Chitin forms microfibrillar configurations in a protein matrix of an organism and their sizes and structure are reliant on the species affiliation of an organism [16,17,18,19]. From the chemical viewpoint, chitin, in close resemblance to cellulose, is an extended-chain copolymer, randomly N-deacetylated [20]. The processability of chitin is limited by its insolubility in many common solvents; therefore, it can be assumed as a biowaste from the food industry [12]. Chitin can be converted into chitosan, which is much more soluble [12,21] and finds applications in several technological fields, mainly in water remediation as an adsorbent or in biomedicine as a drug carrier/antimicrobial agent. Unfortunately, converting chitin into chitosan is a difficult and expensive process [22,23,24].

Chitin contains a high amount of carbon and can be used as a precursor to producing carbon-derived materials with highly interesting parameters, including biochar for electrocatalytic, energetic, and environmental applications [15,25,26]. For example, biochars from crustacean chitin were characterized by a specific surface area ranging from 330 m^2^ g^−1^ to even 2217 m^2^ g^−1^ (chitins activated by different methods) [15,27,28,29,30]. Of these authors, only Zhou et al. [27] studied insect chitin (from the cicada). Recently, some studies reported that 2D (two-dimensional) wrinkled carbon nanosheets can be massively fabricated by using the natural nanofibrous chitin derived from seafood waste. Controllable 2D wrinkled nanosheets from sustainable polymers may have similar or even better properties (depending on the heating rate, calcining temperature, and nitrogen configurations) than materials such as graphene, Mxene, and molybdenum disulfide and would have enormous potential in various applications (in the energy storage/conversion, electrocatalysis) [31,32].

From the presented literature, it can be concluded that there is much scientific research on the potential of biochar from crustacean and other seafood waste chitin. However, there is a knowledge gap about the properties of biochar from insect-derived chitin. Therefore, the aim of these studies was the comprehensive characterization of biochars derived from cricket powder and defatted and deproteinized chitin (chitin as a waste product) from crickets received at various temperatures. The physicochemical characterization of BCs was complemented by an evaluation of the phytotoxicity of BCs in the context of their use in the environment as a soil amendment.

## 2. Results and Discussion

### 2.1. Physiochemical Characterization

**Figure 1:**
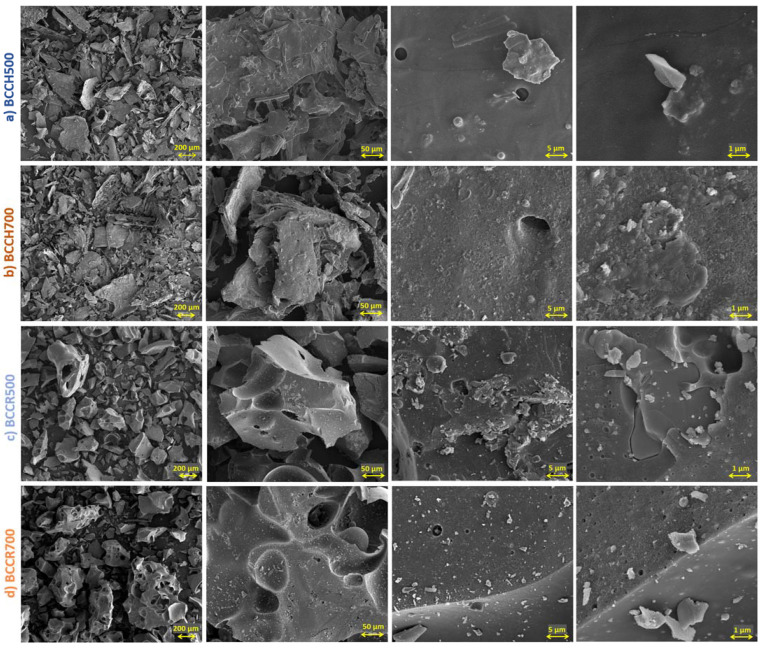
SEM images of BC particles at rows: (**a**) BCCH500; (**b**) BCCH700; (**c**) BCCR500 and (**d**) BCCR700 at different magnification from left to right 100×; 500×; 5000×; 20,000×, respectively.

The morphology of obtained BCs was investigated by SEM analysis (Figure 1). In contrast to the usually highly porous BCs of plant origin [33,34], the presented BCs (Figure 1) are characterized by a densely “packed” solid surface/monolithic type with a non-porous structure with some visible spaces/holes of a few micrometers in diameter. It can be noticed that BCs derived from chitin are composed of thin, irregular-shaped flakes (Figure 1a,b). With increasing the temperature pyrolysis, the BCCH surface becomes more rough with a higher number of tiny, brightly-white particles (typical for inorganic constituents) [35]. In turn, it can be seen that the morphology of BCCRs is slightly different from that of BCCHs. In the particle cross-section, both BCCR500 and BCR700 are in the form of sharp, cracked “rocks” (Figure 1c,d—left side lower magnification), not flakes as in BCCHs. At a higher magnification (Figure 1c,d—right side), the difference in the number of crystal-lites and/or inorganic particles on the surface of BC particles is also visible. Regardless of the pyrolysis temperature, both BCCRs are rich in crystal-lites and inorganic particles. A higher number of inorganic elements such as P, K (existing in salt/oxide form) in BCCRs than BCCHs also was proved by the EDX elemental analysis (Table 1). 

The physiochemical properties of BCs are presented in Table 2. Both cricket- and chitin-based BCs presented similar composition of C, N elements despite the pyrolysis temperature. According to the literature, a part of the protein is chemically bound to the polysaccharide chitin [11] (Barker et al., 1998) and the thermal degradation of chitin prefers the formation of organic N-containing heterocyclic rings (pyrazine, pyridine etc.) compared to ammonia volatilization, which leads to the production of N-doped carbons with potential use in energy storage, fertilization/soil additives and the sorption (e.g., gases, heavy metals and organic compounds) [15,20,26]. Such a high amount of nitrogen in BCs is unusual compared to BCs obtained from other feedstock (from plants waste as well biogas residues, sewage sludge or manure feedstock potentially rich in nitrogen) as well chitin-based BCs received by other researches [36,37,38,39]. In their study, the content of N in BC was in the range from 0.22% (BC from rice husk 800 °C—Jindo) to 5.74 (BC from sewage sludges 500 °C—Zielińska). High N content (8.4%) was reported by Magnacca et al. [15] in BC from snow crab (*Chionoecetes opilio*) pyrolysed at 440 °C. All these studies confirm the decrease in the N content in BC with increasing the pyrolysis temperature. The study by Magnacca et al. [15] additionally showed that a too low pyrolysis temperature (294 °C) decreases the N content of BC (compared to 440 °C and 540 °C).

The natural chemical structure of chitin (composed of N-acetyl-D-glucosamine) causes that the pyrolysis increase BCs’ aromaticity (lower H/C factor), C content and pH value while simultaneously decreasing the O content and hydrophilicity (lower N+O/C factor) [40]. This is observed for both type of BCs in these studies. These are well-known phenomena caused by an intensification of the carbonization degree, the concentration of inorganic constituents (mostly alkali metal salts) with simultaneous cracking of the functional groups [38]. Used feedstock type affected BCs’ pH, EC, and DOC content. BCCHs show a higher amount of pH (over 12 vs. 10) and DOC (especially at 500 °C temperature 63 mg/L vs. 4 mg/L) corresponding to BCCRs. The differences may be due to the chitin extraction procedure with the use of a strong basic NaOH solution (having a strong DOC-leaching ability [41]). The NaOH residues can affect the mentioned BCCH properties. All BCs are characterized by very low porosity parameters, which was also proved by SEM analysis. BCs’ pore structure mostly depends on form feedstock nature and composition. Generally, lignocellulose-based BCs possess a higher S_BET_ than BCs derived from non-lignocellulose feedstock, such as manure/sewage sludge, and high-ash content in BCs also may decrease S_BET_ by pore blockage [42].

**Figure 2:**
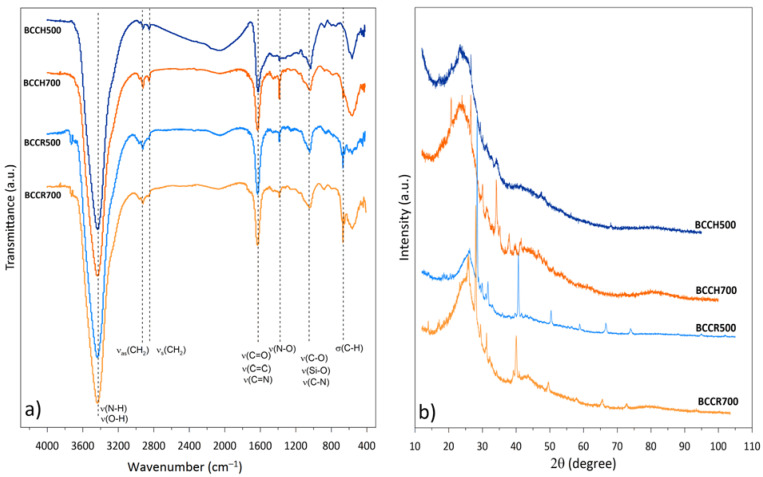
FTIR spectra (**a**) and XRD patterns (**b**) of investigated BC samples.

FTIR spectra (Figure 2 part a) give information about the presence of the functional groups in BC samples. For all BCs (regardless of the pyrolysis temperature and feedstock), similar main bands of diverse intensity can be distinguished.. The most intensive wide band at approximately 3440 cm^−1^ originates from –OH and/or overlaps –NH stretching [43,44]. The double peaks at 2919 cm^−1^ and 2850 cm^−1^corresponded to the asymmetric and symmetric stretching of the aliphatic CH_2_ group, respectively [43]. Intensive band at 1620–1630 cm^−1^ may originate form carbon sp2 double bonds such as the C=O stretching vibration in carboxyl anion, and/or the C=C stretching in aromatic as well the C=N stretching from amino groups and/or N-H bending from amides [26,43,45]. At 1384 cm^−1^ a band from the symmetrical stretching (–NO) nitro groups can be observed with the highest intensity for BCCH700 and the lowest for BCCH500 [44,46].

The wide peak at approximately 1030 cm^−1^ may originate from various overlapping bands as the C–O stretching vibrations in alcohols, phenols, acids, ethers/esters as well as stretching of the Si–O, P–O or C–N [43,45,47]. The band at 670 cm^−1^ designed for the aromatic C–H out-of-plane vibration and/or the Si-O band showed a higher intensity for both samples received in higher temperature. The wide band below 600 cm^−1^ is typically related to the vibrations of the atoms in inorganic constituents (silica, hydroxyapatite, aluminium and iron oxide etc.) [48]. The intensive wide bonds at 530–560 cm^−1^ were described as phosphate [47,49], but due to the overlapping band and the complex BC composition, this region is difficult to identify unequivocally. FTIR spectra proved the presence of the –O, –N-groups as well inorganic constituents in BCs.

From the XRD patterns presented in Figure 2 part b, it can be noticed that the investigated BCs are represented by an amorphous carbon turbostratic structure (randomly oriented graphitic-like micro-crystallites structure) specified by a broad peak at diffraction angles of 23° with other crystallites/sharp peaks of various origin [50]. BCCH500 is characterized by the lower number of crystalline structures (which was confirmed by the SEM analysis) with the most intensive peak from SiO2 (at 26° from 01-089-1961 ICCD reference). With an increase in the temperature pyrolysis, more diverse structure and crystalline phases for BCCH700 appeared. For BCCH700, the most intensive patterns can be assigned to Na_3_PO_4_ (20.5° and 30.4° from 04-008-9362 International Center for Diffraction Data-ICCD reference) and the least intensive are assigned to Na_2_CO_3_ (30° and 34° from 04-009-8706 ICCD reference). FTIR analysis also proved that inorganic phases (phosphates) were present in BCs. In contrast to BCCH500, BCCR500 contains more crystallites with intensity patterns even higher than those obtained in 700 °C. However, the different values of its diffraction angles proved to be a different chemical composition. The most intensive patterns (near 30 and 40°, 50° and 67° from 04-007-9713 ICCD reference) can be distinguished for KCl and the less intensive for K_2_CaP_2_O_7_ (30.5° and 32° from 00-022-0805 ICCD reference). Therefore BCs from crickets powder are more abundant in potassium and phosphate constituents (which are naturally building elements [51] compared to BC derived from chitin (probably due to chitin extraction process using NaOH), which was also confirmed by EDX analysis (Table 1).

The metals content in the analyzed BCs are shown in Table 3. It can be noticed that except for Zn (which is an essential element for the building protein and enzymes of animal organisms and insects are a promising sources of Zn), all BCs are characterized by the other heavy metal content below the limit of detection. Both types of BC (chitin and cricket) are rich in micronutrients (Mg, Ca, Mn, Fe, etc.), while BCCHs contain a few times higher Na content than BCCRs, probably due to the extraction procedure [52].

### 2.2. Phytoxicity of Biochars

**Figure 3:**
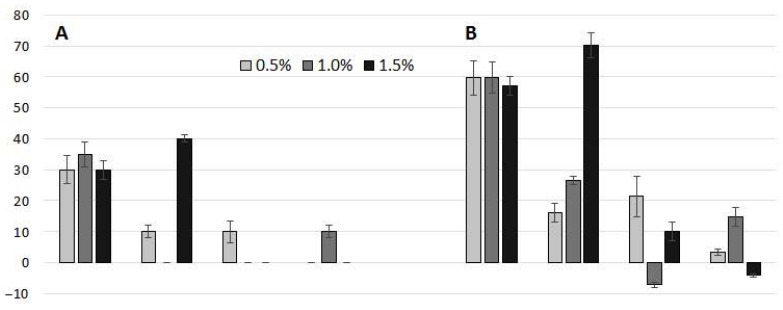
Inhibition of seed germination (**A**) and root growth (**B**) of *Lepidium sativum* in biochar-amended OECD soil at the dose of 0.5, 1.0 and 1.5%. Error bars represent the standard deviation of the mean (*n* = 6) (BCCH—biochar from cricket chitin, BCCR—biochar from micronized crickets).

BCCH500 inhibited germination at 30% (0.5 and 1.5% BCCH dose) and 35% (1.0% BCCH dose). BCCH700 at the 0.5% dose stopped the germination of 10% of the seeds, while at the 1.5% dose it stopped the germination of 40% of the seeds. Biochar from cricket powder (BCCR) completely inhibited the germination of 10% of the seeds at the 0.5% dose of BCCR500 and the 1.0% dose of BCCR700 (Figure 3A).

*L. sativum* roots growing in OECD soil with BCCH500, regardless of dose, were approximately 60% shorter than the *L. sativum* roots growing in the OECD control soil (without BC addition). The negative effect of BCCH700 on root elongation increased with increasing the dose and was 16, 27, and 70% for 0.5, 1.0, and 1.5% doses, respectively. The results of the effects of BCCR on root growth were inconclusive, as both the inhibition (ranging from 3 to 21%) and stimulation of 7 and 4% were recorded for BCCR500—1.0% dose and BCCR700—1.5% dose, respectively (Figure 3B).

Based on the statistical analysis of the correlation of the inhibition indices with the physicochemical characteristics of the tested biochars (Table 4) and the results of studies by other authors [53,54,55], it can be concluded that the toxic factors for *L. sativum* were the increase in the salinity of the substrate and the content of heavy metals in the substrate after adding of tested biochars. The high content of Na in BCs, especially in BCCH 500, was mainly responsible for the increase in salinity (Table 3), which is confirmed by the high correlation coefficients of the inhibition with the high content of this element (Table 4). Similar correlations were shown by Nocentini et al. [55]. The results of these authors also indicate a disturbed ionic balance caused by high concentrations of Mn, B and Zn. The Mn and Zn contents in our all BCs (Table 3) exceeded the toxicity level several times, which is >5, and >150 mg/kg, respectively [55]. However, significant statistical correlations were recorded only between the Mn content and the germination inhibition of *L. sativum* for BCs at a dose of 1.5%.

An additional negative effect on germination and root growth is related to the high pH of the biochar [54,55,56]. The studied BCs were characterized by high pH (Table 2), but statistical calculations did not show significant correlations of this parameter with the inhibition (Table S1). Table S2. Crystalline phases (with ICCD codes) in BCCH700. Table S3. Crystalline phases (with ICCD codes) in BCCR500. Table S4. Crystalline phases (with ICCD codes) in BCCR700. Some inconclusive results may be the effect of the interaction of the toxic factors with the effect of the germination and root growth stimulation caused by nutrients contained in BCs such as N, P, K [56]. The germination stimulation effect is confirmed by the negative values for the correlation of the germination inhibition with the nitrogen content of BCs (for 1.5% dose) and ash content with the root growth inhibition (for 1% dose). In conclusion, it can be stated that the use of biochar obtained from chitin to improve soil properties is associated with a risk of toxicity to plants, but it can also have a stim-ulating effect depending on the type of BC used and the dose. Hence, this is-sue requires more extensive field studies. Figures S1–S4: XRD patterns of BCCH500, BCCH700, BCCR500 and BCCR700.

## 3. Materials and Methods

### 3.1. Fractionation of Cricket Powder

Frozen crickets (*Acheta domesticus*) were dried in a freeze-drying process and then ground. In order to obtain waste chitin, the ground cricket powder was defatted and deproteinized. Fat content was removed via extraction in petroleum ether using AOCS protocol 991.36 [57]. Further lipid was extracted from the solid with hexane [58] and the modified method by Babiker et al. [59].

A protein fraction was extracted from defatted cricket powder-based on Kim and others’ method [60]. The dry defatted powder was dispersed in ultrapure water, adjusted to pH 9.5 using 1.0 M sodium hydroxide solution, and agitated for 2 h. The insoluble residue (waste chitin) was separated by centrifugation at 35,267× *g* for 15 min. The process was repeated three times.

### 3.2. Chemicals

Ultrapure 65% HNO_3_ and 30% HCl were purchased from Ciech Trading (Poland) and used for sample digestion. The ICP multi-element standard solution IV (1.11355.0100) was received from Merck (Darmstadt Germany).

### 3.3. Biochar Preparation

Micronized cricket powder (CR) and defatted and deproteinized cricket chitin (CH) were used as feedstock for BC production (BCCR and BCCH). The feedstock was pyrolyzed using the retort furnace (PRC 168X380/90g Czylok, Jastrzębie-Zdrój, Poland) under two temperatures: 500 °C and 700 °C for 1 h with 1.5 L/min of N_2_ flow.

### 3.4. Physiochemical Characterization

The pH and electrical conductivity (EC) of BC/feedstock water extracts were measured using a digital multi-parameter HQ430d Benchtop Single Input (HACH, Loveland, Colorado, CO, USA). The extracts were prepared by shaking of 2 g solid sample with 20 mL of milliQ water for 24 h; afterwards, samples were centrifuged and filtered (PTFE hydrophilic 0.45 μm syringe filter). The total organic carbon content (TOC) in solid samples and the dissolved carbon content (DOC) in water extracts (prepared as described above) were determined using a TOCVCSH SHIMADZU (Kyoto, Japan) with a Solid Sample Module SSM-5000. The content of carbon, hydrogen, and nitrogen in analyzed samples were determined using CHN equipment Perkin–Elmer 2400 (Akron, Ohio, OH, USA). The ash content of samples was determined following the ASTM D 3176 standard method by combustion of dry samples at 760 °C for 6 h [37]. Nitrogen adsorption/desorption isotherms were measured at 77 K using a volumetric adsorption analyzer Micromeritics ASAP 2405 (Atlanta, Georgia, GE, USA). The standard Brunauer–Emmett–Teller method was used to determine the specific surface area (SBET) [61]. The total pore volume (V_p_) was estimated from single-point adsorption at *p*/*p*_0_ = 0.99. FTIR spectra of the samples were recorded by the Nicolet 8700A Thermo Scientific (Waltham, Massachusetts, MA, USA) spectrometer at RT over the 4000–400 cm^−1^ range at the resolution of 4 cm^−1^.

The sample SEM images were received from a Quanta scanning electron microscope SEM; FEI Quanta 3D FEG (USA), equipped with an energy dispersive X-ray spectrometer (EDX), working at 20 kV. The XRD analysis (see Supplementary Materials) was performed using a high-power X-ray diffractometer Empyrean, PANalytical (GH Eindhoven, Netherlands). The diffraction patterns were recorded in the 2θ range from 5° to 95° using CuKα radiation.

The total metal concentration was analyzed using a microwave oven (Start D, Microwave Digestion System, Milestone, Sorisole (BG), Italy) via a wet method in a mixture of 65% nitric acid (9 mL) and 30% hydrochloric acid (3 mL) with a total volume pof 12 mL to 0.2 g BCs or 0.5 g feedstocks. The metal content after acid mineralization was determined using emission spectrometry on ICP-OES Model iCAP 7400 Duo, Thermo Scientific (Waltham, Massachusetts, MA, USA).

### 3.5. Phytotoxicity Test

Phytotoxicity of the BCCH and BCCR samples was assessed by the commercial solid toxicity bioassay, Phytotoxkit F™, with *Lepidium sativum*. Artificial OECD soil was used as a reference soil, to which BCCH and BCCR were added at a dose of 0.5, 1.0, and 1.5% (based on dry weight).

## 4. Conclusions

Regardless of the pyrolysis temperature, the BCs were characterized by a densely “packed” type of solid surface/monolith with a non-porous structure that ranged from 0.05 to 0.22 m^2^/g. Both chitin and ground cricket BCs were characterized by the high N content (ranging from 9.4% to 11.8%) and a high pH. This demonstrates their high potential for use in energy storage technology and for the fertilization/improvement of the soil’s physicochemical properties. Based on the XRD analysis, BCs were characterized by amorphous carbon turbostratic structure, and a randomly oriented graphitic-like micro-crystallite structure. The FTIR spectra of BCs confirmed the presence of various oxygen and nitrogen functional groups on their surface. BCCRs are richer in potassium and phosphate components (which are naturally building elements) than BCCHs, which was also confirmed by the EDX analysis. BCCHs added to soil at rates from 0.5 to 1.5% significantly reduced germination of *Lepidium sativum*. However, the stimulation of *L. sativum* root elongation was also observed with the BCCR500 1.0% and the BCCR700 1.5%. The immediate short-term phytotoxicity shown in Phytotoxkit F™ may be of some limitation to the use of chitin BCs as a soil additive. However, for a definitive conclusion, toxicity studies under field conditions would need to be conducted. The balance of benefits and losses may prove positive over a longer period of time. This research shows that the standard methods in this type of research may be insufficient and better methods of preliminary preparation and treatment of this type of material should be searched for.

## Figures and Tables

**Table 1:** BC elemental analysis from SEM–EDX data.

Element wt%	BCCH500	BCCH700	BCCR500	BCCR700
C	69.49 ± 0.57	67.03 ± 1.86	71.58 ± 0.90	70.65 ± 2.11
N	9.84 ± 0.62	8.00 ± 0.37	11.80 ± 2.18	11.15 ± 0.70
O	13.28 ± 0.57	15.69 ± 1.81	9.23 ± 0.93	9.33 ± 2.45
Na	4.10 ± 0.13	4.83 ± 0.61	1.19 ± 0.36	1.24 ± 0.18
Ca	1.28 ± 0.42	1.80 ± 0.45		
K		0.84 ± 0.29	2.87 ± 0.67	4.17 ± 1.48
P			2.04 ± 0.55	2.06 ± 0.46

**Table 2:** The physicochemical properties of feedstock and derived BCs.

Value	Ash	pH	EC	TOC	DOC	C	H	N	O	O/C	(O+N)/C	H/C	S_BET_	Yield
	(%)		(µS/cm)	(%)	(mg/L)	(%)	(%)	(%)	(%)				(m^2^/g)	(%)
Chitin	9.4	10.13	11060	47.3	nd	46.2	6.3	10.2	27.9	0.45	0.64	1.62	nd	-
BCCH500	15.2	12.2	8.8	60.1	63.4	65.7	2.3	9.8	7.0	0.08	0.21	0.41	0.05	25.1
BCCH700	16.4	12.4	12.1	53.4	9.6	70.3	1.2	9.4	2.7	0.03	0.14	0.21	0.17	24.5
Cricket	3.8	5.5	5560	47.6	nd	52.7	7.9	10.6	24.9	0.35	0.53	1.79	nd	-
BCCR500	17.9	8.7	4.2	57.6	4.0	63.6	2.2	11.2	5.1	0.06	0.21	0.42	0.22	22.9
BCCR700	16.2	10.8	2076.7	52.2	3.3	69.1	1.0	10.6	3.1	0.03	0.17	0.17	0.04	22.3

Where: ash = ash content; pH = pH value; EC = electrical conductivity; TOC = total organic carbon; DOC = dissolved organic carbon; C, H, N, O = element content and O% calculated by difference 100−(C+H+N+Ash); O/C, (O+N)/C, H/C = molar ratios; S_BET_ = specific surface area, yield = pyrolysis yield.

**Table 3:** Metals (heavy metal: Zn, Cu, Pb, Ni, Co, Cd, Cr and metal mineral nutrients Fe, Na, Ca, Mg, Mn, B) content [mg/kg] in BCs sample.

Metal [mg/kg]	BCCH500	BCCH700	BCCR500	BCCR700
Na	41,792.36	33,476.92	14,177.70	9614.68
Ca	16,028.90	17,858.92	5475.49	4301.13
Mg	3298.81	3644.61	3419.12	2120.66
Zn	888.74	936.21	892.77	630.40
Fe	476.31	379.71	534.31	289.26
Mn	274.75	294.43	159.31	117.17
B	50.86	62.87	21.45	100.08
Cu	37.83	40.46	88.85	26.24
Pb	<LOD	<LOD	<LOD	3.66
Ni	<LOD	<LOD	<LOD	<LOD
Co	<LOD	<LOD	<LOD	<LOD
Cd	<LOD	<LOD	<LOD	<LOD
Cr	<LOD	<LOD	<LOD	<LOD

**Table 4:** Relationship between physicochemical properties and metals content in BCs and Phytotoxkit endpoints (statistically significant coefficient: * *p* < 0.1; ** *p* < 0.05).

	Inhibition of Seed Germination			Root Growth Inhibition		
	0.5%	1.0%	1.5%	0.5%	1.0%	1.5%
ASH%	−0.545	−0.821	−0.560	−0.541	−0.946 *	−0.482
pH	0.410	0.454	0.868	0.343	0.839	0.783
EC	0.507	0.099	0.976 **	0.401	0.552	0.986 **
TOC	0.876	0.619	0.135	0.904 *	0.401	0.269
DOC	0.944 *	0.938 *	0.491	0.955 **	0.896	0.528
C%	−0.403	−0.197	0.438	−0.466	0.207	0.288
H%	0.749	0.427	−0.006	0.779	0.174	0.148
N%	−0.412	−0.300	−0.956 **	−0.324	−0.752	−0.894
O%	0.857	0.753	0.037	0.906 *	0.469	0.149
Cu	0.010	−0.429	−0.368	0.020	−0.627	−0.220
Zn	0.589	−0.037	0.640	0.510	0.195	0.754
Al	0.690	0.379	0.983 **	0.603	0.762	0.991 **
B	−0.465	0.083	−0.038	−0.452	0.187	−0.204
Ba	0.687	0.435	0.981 **	0.604	0.812	0.976 **
Bi	0.927 *	0.958 **	0.404	0.950 **	0.866	0.444
Ca	0.636	0.324	0.993 **	0.544	0.734	0.995 **
Fe	0.597	0.125	0.013	0.603	−0.049	0.180
Ga	0.927 *	0.958 **	0.404	0.950 **	0.866	0.444
In	0.802	0.598	0.922	0.739	0.893	0.928 *
Mg	0.527	−0.112	0.623	0.444	0.134	0.735
Mn	0.676	0.297	0.975 **	0.584	0.685	0.998 **
Na	0.849	0.584	0.905 *	0.786	0.856	0.932 *
Sr	0.595	0.323	0.998 **	0.503	0.747	0.985 **

## Data Availability

All data generated or analyzed during this study are included in this published article (and it is in Supplementary Information files).

## Supplementary Materials

The following supporting information can be downloaded at: https://www.mdpi.com/article/10.3390/molecules27228071/s1, Figure S1: XRD patterns of BCCH500; Figure S2: XRD patterns of BCCH700; Figure S3: XRD patterns of BCCR500; Figure S4: XRD patterns of BCCR700; Table S1: Crystalline phases (with ICCD codes) in BCCH500; Table S2: Crystalline phases (with ICCD codes) in BCCH700; Table S3: Crystalline phases (with ICCD codes) in BCCR500; Table S4: Crystalline phases (with ICCD codes) in BCCR700.

## Sample Availability

Samples of the compounds are not available from the authors.
