# Supplementary material for: Physicochemical Characteristics of Biochar from Waste Cricket Chitin (Acheta domesticus)
Source: Molecules. 2022 Nov 21;27(22):8071. doi: 10.3390/molecules27228071 (PMC9692431; doi:10.3390/molecules27228071)
Supplement: Supplementary file 1 [file molecules-27-08071-s001.zip › molecules-1993892-supplementary.pdf]

SUPPORTING INFORMATION

Physicochemical Characteristics of Biochar from Waste Cricket Chitin (*Acheta domesticus*)

Krzysztof Różyło [1,*], Katarzyna Jędruchniewicz [2], Patrycja Krasucka [2], Wojciech Biszczak [1] and Patryk Oleszczuk [2]

[1] Department of Herbology and Plant Cultivation Techniques, University of Life Sciences in Lublin, Lublin, Poland
[2] Department of Radiochemistry and Environmental Chemistry, Faculty of Chemistry, Maria Curie-Skłodowska University, Lublin, Poland
* Correspondence: krzysztof.rozylo@up.lublin.pl; Tel./Fax: +48-81-445-66-69

Data from XRD analysis

Table S1. Crystalline phases (with ICCD codes) in BCCH500.

Lp	Ref. Code	Compound Name	Chemical Formula	SemiQuant [%]
1	01-089-1961	Silicon Oxide	SiO_2	

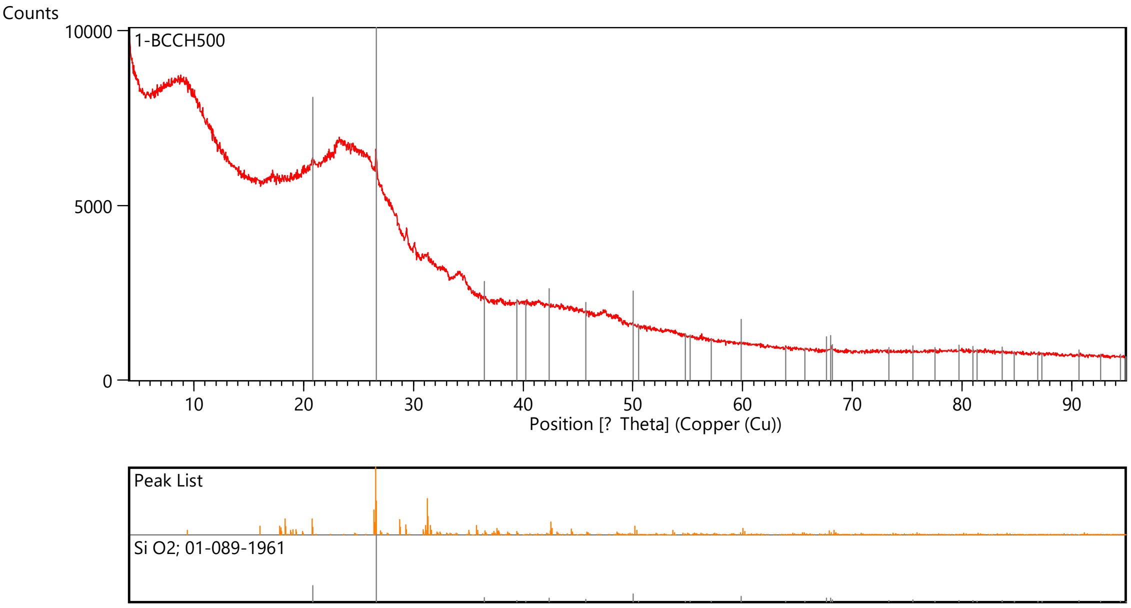

Figure S1. XRD patterns of BCCH500.

Table S2. Crystalline phases (with ICCD codes) in BCCH700.

Lp	Ref. Code	Compound Name	Chemical Formula	SemiQuant [%]
1	04-009-8706	Sodium Carbonate	$Na_2(CO_3)$	20(2)
2	04-008-9362	Sodium Phosphate	$Na_3(PO_4)$	80(20)

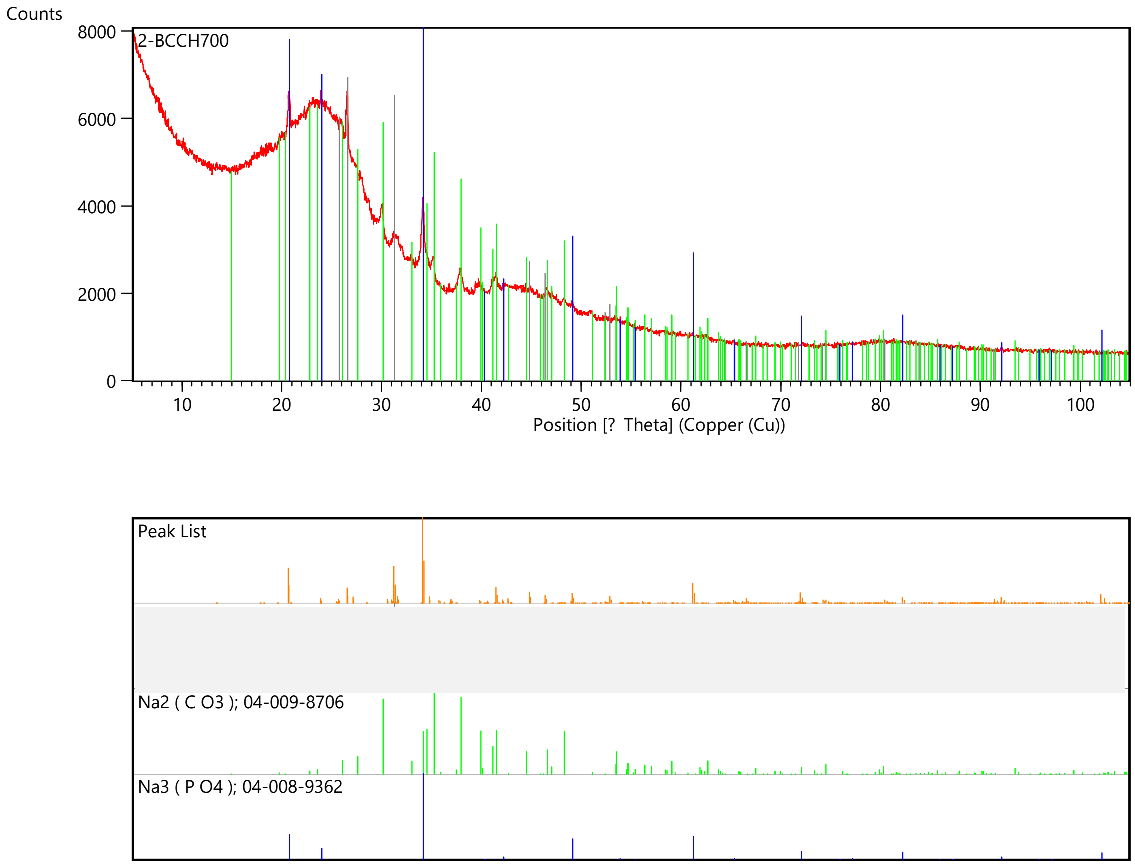

Figure S2. XRD patterns of BCCH700.

Table S3. Crystalline phases (with ICCD codes) in BCCR500.

Lp	Ref. Code	Compound Name	Chemical Formula	SemiQuant [%]
1	04-007-9713	Potassium Chloride	KCl	52.0(4)
2	00-035-0819	Potassium Phosphate	KPO_3	11(1)
3	00-022-0805	Potassium Calcium Phosphate	$K_2CaP_2O_7$	22(1)
4	04-017-8676	Potassium Sodium Phosphate	K2Na3P3O10	16(1)

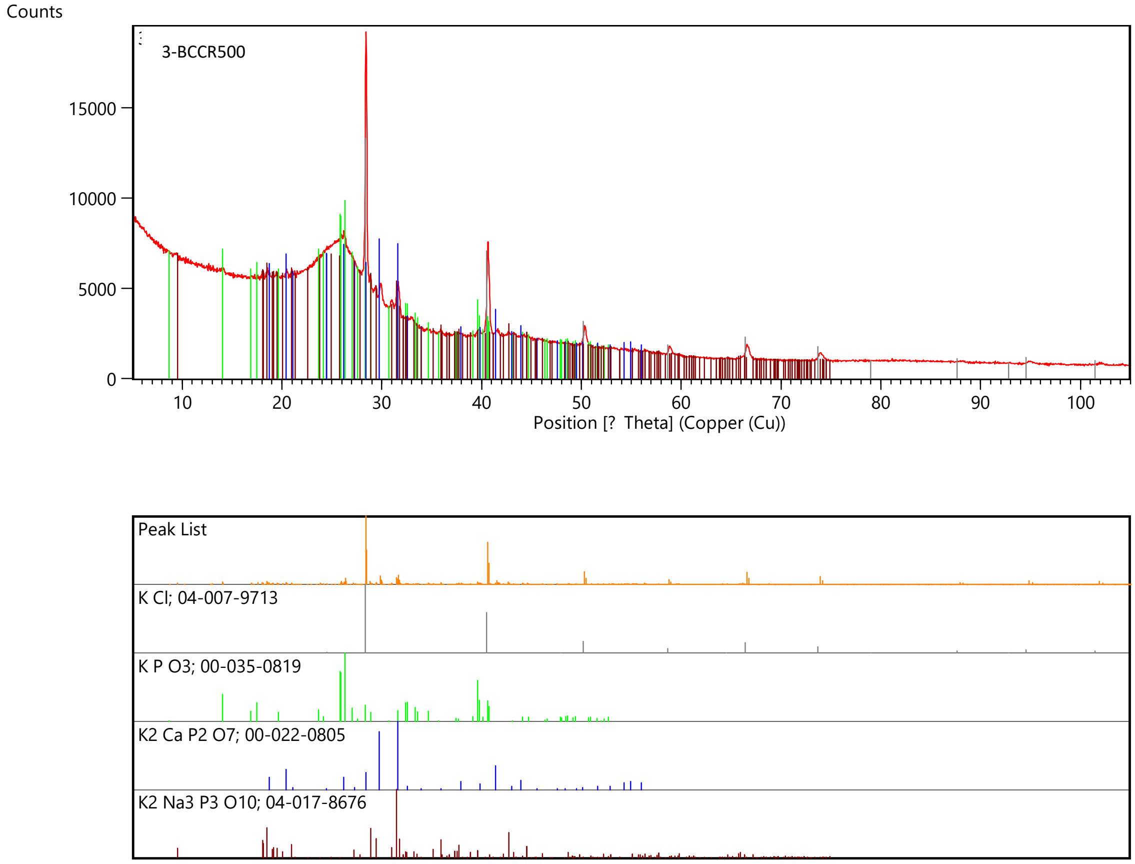

Figure S3. XRD patterns of BCCR500.

Table S4. Crystalline phases (with ICCD codes) in BCCR700.

Lp	Ref. Code	Compound Name	Chemical Formula	SemiQuant [%]
1	04-007-9713	Potassium Chloride	KCl	38(1)
2	00-035-0819	Potassium Phosphate	KPO_3	44(1)
3	00-022-0805	Potassium Calcium Phosphate	$K_2CaP_2O_7$	18(1)

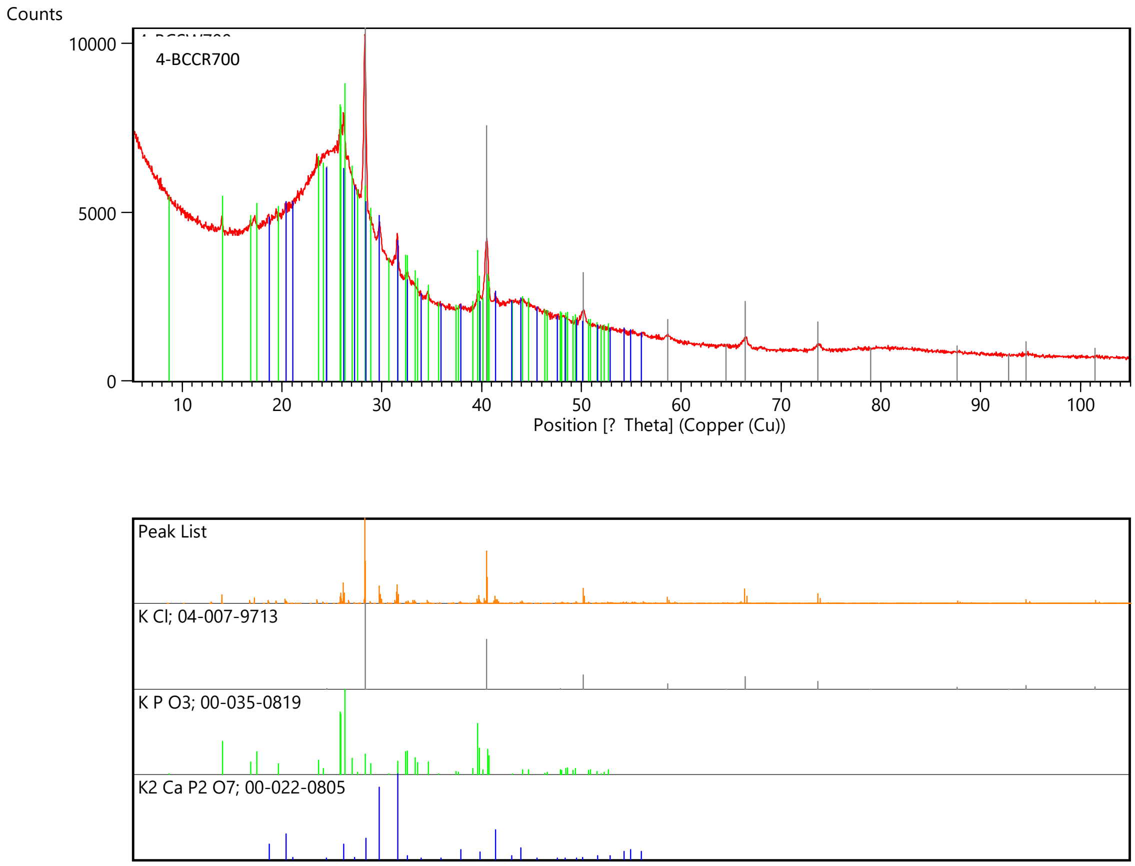

Figure S4. XRD patterns of BCCR700.
